# Best practices for analyzing large-scale health data from wearables and smartphone apps

**DOI:** 10.1038/s41746-019-0121-1

**Published:** 2019-06-03

**Authors:** Jennifer L. Hicks, Tim Althoff, Rok Sosic, Peter Kuhar, Bojan Bostjancic, Abby C. King, Jure Leskovec, Scott L. Delp

**Affiliations:** 1 Department of Bioengineering, Stanford University, Stanford, CA USA; 2 Paul G. Allen School of Computer Science & Engineering, University of Washington, Seattle, WA USA; 3 Computer Science Department, Stanford University, Stanford, CA USA; 4 Azumio, Inc., Redwood City, CA USA; 5 Department of Health Research and Policy, Stanford University School of Medicine, Stanford, CA USA; 6 Stanford Prevention Research Center, Department of Medicine, Stanford University School of Medicine, Stanford, CA USA; 7 Chan Zuckerberg Biohub, San Francisco, CA USA; 8 Department of Mechanical Engineering, Stanford University, Stanford, CA USA

**Keywords:** Data mining, Statistical methods, Health sciences

## Abstract

Smartphone apps and wearable devices for tracking physical activity and other health behaviors have become popular in recent years and provide a largely untapped source of data about health behaviors in the free-living environment. The data are large in scale, collected at low cost in the “wild”, and often recorded in an automatic fashion, providing a powerful complement to traditional surveillance studies and controlled trials. These data are helping to reveal, for example, new insights about environmental and social influences on physical activity. The observational nature of the datasets and collection via commercial devices and apps pose challenges, however, including the potential for measurement, population, and/or selection bias, as well as missing data. In this article, we review insights gleaned from these datasets and propose best practices for addressing the limitations of large-scale data from apps and wearables. Our goal is to enable researchers to effectively harness the data from smartphone apps and wearable devices to better understand what drives physical activity and other health behaviors.

## Introduction

Commercial wearable devices and smartphone apps for monitoring health-related behaviors have proliferated rapidly. In 2013, 69% of U.S. adults reported tracking one or more health indicators, such as weight or exercise, and 21% of those used technology, such as an app or device,^1^ while others monitored these health indicators “in their heads” or on paper. The mobile health market is projected to grow to $500 billion worldwide by 2025.^2^ Apps and devices are available to monitor a wide range of health behaviors and indicators, such as physical activity, sedentary behavior, weight, diet, heart rate, blood pressure, and sleep. Data can be collected via self-report in the app, through integrated sensors (e.g., accelerometers), or through integration with other devices, like digital scales and blood pressure cuffs.

Analyzing the data generated by commercial wearables and apps has the potential to alter how we study human behavior and how we intervene to improve health. These datasets are orders of magnitude larger than traditional research studies and can be accessed by researchers at relatively low cost. Since much of the data are collected automatically, they can reveal behavior in the natural environment and reach individuals who do not typically enroll in research studies and who have not altered their behavior because they are being monitored in a research study. Modifiable health behaviors like physical activity,^3^ sedentary behavior,^4^ and sleep^5^ have a significant impact on many aspects of cardiovascular, musculoskeletal, and mental health, but until the advent of modern wearables we have had limited tools to study these interrelated behaviors at scale. Changing health behaviors has been challenging,^6^ but the large-scale data from apps and wearables can help uncover the environmental, social, and personal factors that motivate healthy behaviors and identify new ways to promote sustained behavior change.

In spite of the promise of mobile apps and devices and the massive amounts of data they are collecting, analysis has been limited by several challenges. Effectively analyzing these data requires expertise in both data science and health behaviors, and few researchers are dually trained, often making collaboration and communication between disciplines difficult. A lack of trust also presents a major challenge: consumers question if privacy will be protected, researchers question if results are valid, and companies question how academic partnerships will affect their business.

These challenges motivate this article. Our goal is to foster confidence in using large-scale datasets from consumer apps and wearables to better understand the relationships among physical activity and other health behaviors and health outcomes. We hope this article encourages data sharing between academia and industry by highlighting productive examples. We also hope to bridge the divide between health researchers and data scientists by establishing a common knowledge base. We first highlight several example studies that have used observational data from consumer apps and wearable devices to study human health. From these studies, we identify both novel insights and common challenges. We outline best practices for analyzing data from consumer apps and wearables and conclude with a summary of areas where additional research is needed.

This article focuses on studies that have analyzed large-scale data (e.g., thousands of individuals) collected through routine use of commercial wearables and smartphone apps by consumers. We include apps and devices that monitor health behaviors and indicators, including physical activity, weight, diet, sleep, sedentary behavior, blood pressure, and heart rate. There is excellent research using commercial devices in small scale studies^7,8^ and studies that have focused on validating the use of these devices in a variety of populations.^9,10^ This work is valuable but is not the focus of the present article.

## Highlights from the literature: insights and challenges

**Fig. 1:**
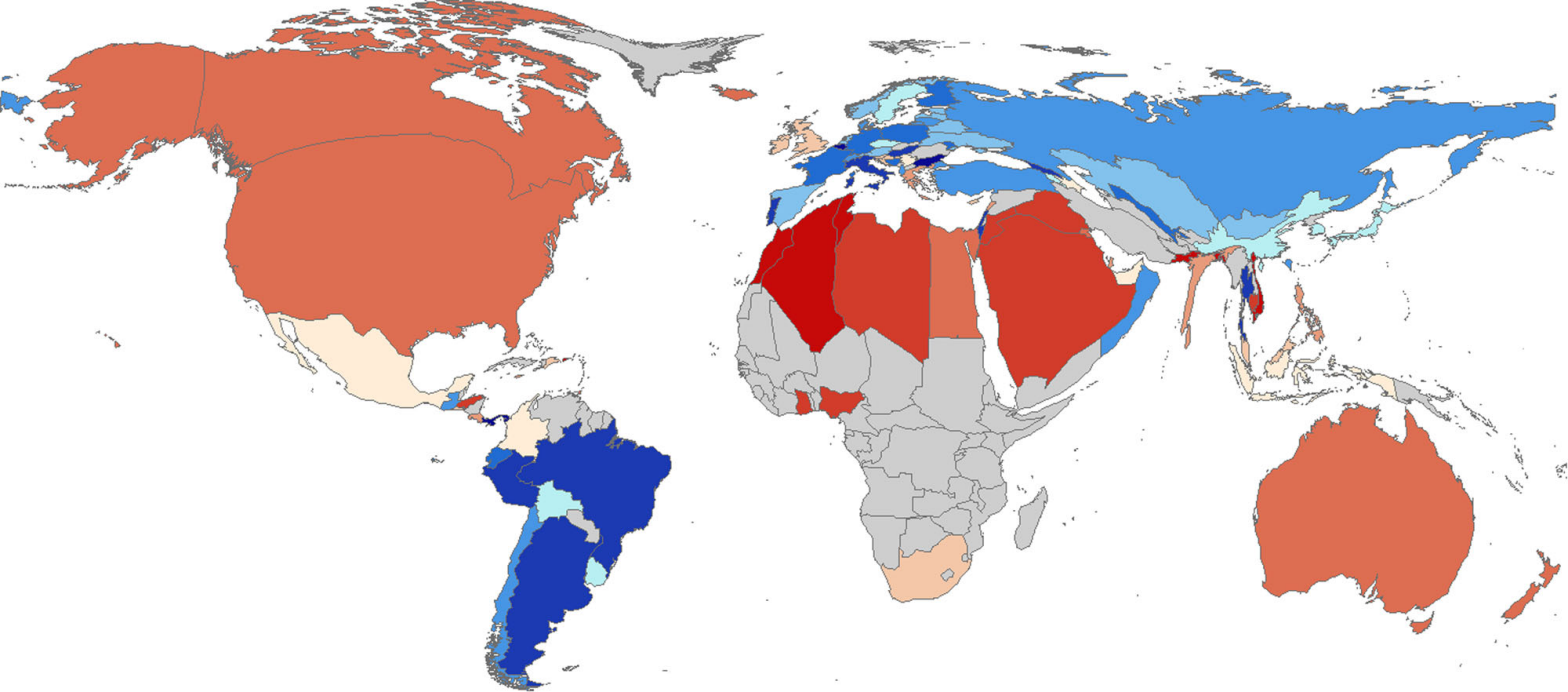
Datasets from apps and wearables are helping researchers identify novel worldwide trends in activity and health. Our team has analyzed data from 717,527 users of the Argus app for tracking physical activity and other health metrics.^11^ This analysis revealed worldwide inequality in levels of physical activity that varied from country to country. In the map, country area is scaled by the country’s obesity rate, as calculated from the app-reported BMI of users. The countries are shaded according to activity inequality, where warm colors (reds and oranges) indicate high levels of activity inequality (some people are very active and some people are minimally active) and cool colors (blues) indicate low levels of activity inequality (individuals within the country get similar levels of activity). Countries with larger than normal areas (indicative of high obesity) also tend to be shaded with warm colors (indicative of high activity inequality). The map was generated using the Scape Toad software^63^ and the world borders dataset from the Thematic Mapping API^64^

Several studies have used data from commercial apps and wearables to characterize health behaviors and their potential influence on health indicators, like weight and cognitive performance. For example, our group has analyzed data from over 700,000 users of a smartphone app (Argus, Azumio, Inc.) for tracking physical activity.^11^ We analyzed minute by minute step counts estimated automatically using the smartphone’s onboard inertial measurement unit (IMU) in individuals from over 100 different countries. This analysis revealed that inequality in how physical activity is distributed between individuals in a country (i.e., the Gini coefficient^12^ applied to step counts) is a stronger predictor of obesity rates than average activity levels in a country (Fig. 1). By connecting activity tracking results to a database of city walkability scores, we also showed that higher walkability scores are associated with lower activity inequality in U.S. cities.

Sleep is another important and modifiable health behavior. Walch and colleagues^13^ analyzed sleep schedule, light exposure, and other data from 8000 users of a free sleep-tracking smartphone app. They used these data to help untangle how social factors, light exposure, and the circadian rhythm influence sleep, demonstrating that social pressures delay bedtime, attenuating or overriding biological pressure for sleep. Althoff et al.^14^ connected wearable-determined sleep metrics with performance measured through the individual’s interaction with a search engine (e.g., keystroke time and time to click on a resulting page), showing that two consecutive nights with less than 6 h of sleep is associated with decreased performance for a period of 6 days.

Variability in blood pressure is predictive of future cardiovascular disease and morbidity, but has been challenging to characterize with traditional studies, particularly in real-world settings (as opposed to clinical settings which can influence vital signs). Kim et al.^15^ analyzed blood pressure readings from over 50,000 individuals (with 17 million measurements) using a wireless blood pressure monitor. They characterized blood pressure variability and how it changes with season of the year, day of the week, and time of day, for example, showing that variability is higher during weekdays, particularly for females. Researchers also have quantified how holidays affect weight gain using data from digital scales^16^ and examined how factors like geographic location and body mass index (BMI) are related to the taste profile (salty, sweet, etc.) of the meal an individual selects and reports in a diet tracking app.^17^

Many apps include features like a social network, challenges, or competitions, which are intended to motivate healthy behavior and usage of the app or device. Researchers have used large-scale app data to understand how these features influence physical activity and other behaviors. Aral and Nicolaides^18^ analyzed exercise patterns in a global social network of 1.1 million runners, demonstrating “contagion” of exercise that varies based on gender and relative levels of activity. For example, they found that both men and women influence the activity levels of men, but only women influence other women. Althoff et al.^19^ used the dataset from the Argus smartphone app to identify a natural experiment and show that forming new social connections in the app increases daily step count by an average of ~400 steps per day. In this dataset, women receiving friendship requests from other women recorded greater increases in activity than women who received requests from men or men who received requests from either gender. The Argus app also includes games where groups of people compete to record the greatest number of steps over a specified period of time; these competitions were found to increase physical activity by 23% during the time of the competition.^20^ The success of the competition varied according to the composition of the group. Competitions where teams had an even gender split had the largest increases in activity. Wang et al.^21^ analyzed data from 10 million users of the BOOHEE app to determine the influence of a social network on weight status, and found that users were more likely to lose weight when they had more network friends of the opposite sex. Pokemon Go experienced a period of widespread usage during which Althoff et al.^22^ showed that engaged Pokemon Go users increased their activity by nearly 1500 steps per day (a 25% increase). This work demonstrates the promise of combining multiple datasets—the researchers quantified the effect of Pokemon Go on activity by combining data from internet searches (to predict who was using the Pokemon Go app) and a smartwatch (to quantify physical activity).

Large-scale data have also enabled researchers to build predictive models of health and behavior. For example, Shameli et al.^20^ developed a model to predict whether a competition will increase physical activity of participants, reporting an area under the receiver operating characteristic curve (known as AUC; a common measure of model classification accuracy) of 0.75 for a model using factors such as participant demographics and previous amounts of physical activity. Althoff et al.^19^ also built a model to predict whether a future in-app social network link will lead to increased physical activity, with an AUC of 0.78 for a model using similar types of features. Kurashima et al.^23^ built a model to predict actions (e.g., going for a run, drinking water, recording weight, going to sleep) and the timing of actions in the Argus data, along with a similar model using data from Under Armour’s MyFitnessPal app (with data from 12 million users). The models predicted whether an action would occur with 50–60% accuracy and predicted action timing with a mean absolute error of <150 min, which improved on the performance of existing models by up to 150%.

Researchers have used these large-scale datasets to identify clusters of similar users based on their behavior and health status. Serrano and colleagues^24,25^ mined data from approximately 1 million users of the LoseIt! App to identify subgroups based on weight loss. They identified what they categorized as occasional, basic, and “power” users of the app, with the power users showing the greatest weight loss. With their MyHeartCounts app, McConnell et al.^26^ studied the relationship between physical activity and self-reported cardiovascular disease in nearly 50,000 individuals. They found that a subgroup of individuals who transitioned frequently between activity and inactivity had similar self-reported levels of cardiovascular disease as a second group with higher overall activity levels but fewer transitions.

For apps and devices to successfully change behavior, users must engage for a sustained period of time. Researchers have analyzed these datasets to determine who engages, how users engage, and factors that promote engagement. Lin et al.^27^ have used the Azumio dataset to explore patterns and cycles of app usage. They found that the majority (75%) of users re-engaged with the app after extended periods of dormancy and that upon re-engagement, user behavior and health status (e.g., weight levels) appeared as they did when they were first using the app, rather than picking up where they left off. In addition, Park et al.^28^ examined the traits of individuals who share their exercise app data often and for an extended period; these individuals tended to have a fitness-focused network of friends and, surprisingly, less popular tweets. Sperrin et al.^29^ showed that smart scale users who weighed themselves often were more likely to lose weight and that users were more likely to weigh themselves after a recent reduction in weight, suggesting that further research is needed to understand whether close monitoring drives weight loss or vice versa.

### Summary of insights

Analyzing data from consumer users of apps and wearables has allowed researchers to characterize health indicators, such as blood pressure variability,^15^ and points to promising new avenues of research, such as focusing physical activity interventions on the activity poor segment of a population.^11^ We also see that external factors, such as the walkability of a city^11^ and social pressures preventing sleep,^13^ can have a strong influence on health behaviors. This supports the findings of previous, more traditional studies and with the larger subject numbers in consumer app datasets, we can quantify associations between external factors and health behaviors across different age and gender groups. Analyzing these large datasets can also help improve the design of apps and wearables. Multiple studies show that social influence and gamification are associated with increases in healthy behavior^18–20,22^ and that gender is an important covariate. Predictive models could help apps that promote physical activity become more effective by, for example, guiding friendship recommendation algorithms^19^ or creating effective groups for activity competitions.^20^ Users’ goals and how individuals use health apps can be variable, but the data available about a user’s interactions with an app (even in the first few days) can predict much of this variation.^24,25,27–29^ Using this knowledge, app designers could create more engaging and personalized apps that are more effective in achieving behavior change.

### Summary of challenges

Reviewing this literature and reflecting on our own experience analyzing these large-scale datasets reveal several common challenges and potential sources of error. Since these datasets are not generated to test a specific hypothesis, the data is almost always “messy” and difficult to analyze for a variety of reasons. Measurement error can arise from the inaccuracy of sensors in estimating quantities of interest (e.g., steps or sleep) and such errors can be systematic (e.g., wearables for tracking physical activity can systematically underestimate steps for slow walking^30^). Kim and colleagues^15^ use a device that has been validated according to established protocols. Some of the studies analyzing sleep, steps, and physical activity, conduct their own experiments,^26^ cite validation studies in the scientific literature,^11^ and/or compare values and trends to previous literature and datasets.^11,13,14^ Missing data is another challenge, since individuals do not always wear their device or carry their phone. Selection bias can also occur, as individuals who use apps and wearables may not represent the gender, age, geographic location, socioeconomic status, and/or race/ethnicity of the population of interest. Most studies acknowledge these issues and several conduct sensitivity or robustness testing. Handling very large datasets can also be challenging with a typical computer and traditional methods, particularly for researchers with limited expertise in machine learning and data science. Forming academic-industry data sharing partnerships has also been a major limiting factor in the number of studies conducted thus far.

## Best practices: an iterative process

**Fig. 2:**
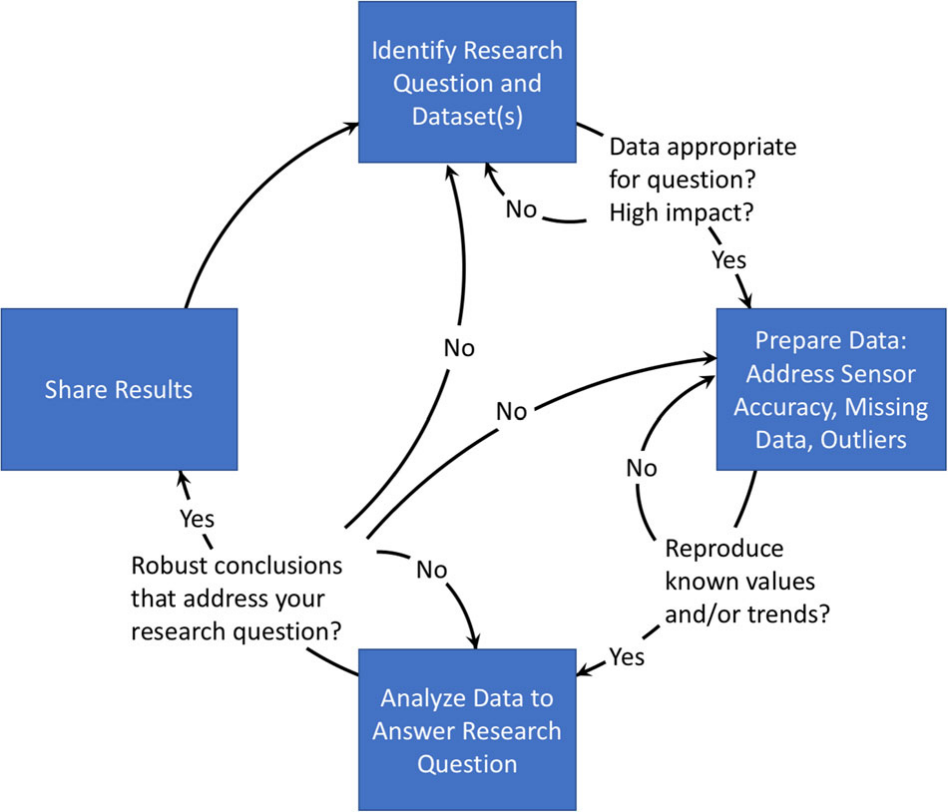
Overview of best practices for analyzing large-scale physical activity and health behavior datasets from commercial smartphone apps and wearable devices. The process is highly iterative, as indicated by the arrows flowing in both directions. By sharing results, along with data and software tools, your work can help inspire new research, completing the circle

In response to the challenges described above, we propose the following iterative process for designing a study, analyzing the data, and reporting the results (Fig. 2).

### Step 1: identify your research question and dataset(s)

The first step is to identify your research question and the data needed to address the question. This phase is vital to ensure that the work will be high impact, advance science, and lead to robust conclusions. By approaching a dataset of interest with specific questions or hypotheses, investigators reduce the risk of Type I (false positive) errors. Combining expertise from data science and health research can help researchers navigate the planning phase successfully.

You should begin by identifying a question that will have an impact on health, based on gaps in knowledge. For example, some of the key needs include quantifying geographic differences and the role of environmental factors in health behaviors and delineating the relationships between multiple health behaviors (e.g., physical activity, sedentary behavior, and sleep). A “naïve” data mining approach (i.e., searching for correlations, clusters, etc. without a specific research question in mind) is almost never useful. Without a research question to frame the analysis, it is challenging to identify interesting and high-impact correlations. This approach also lacks an experimental design to establish internal and external validity (see Step 5).

For observational datasets, establishing irrefutable causal relationships is typically not possible; however, the data often contain natural experiments that allow for causal analysis. Correlational analysis of observational data has helped transform science and medicine, for example, linking tobacco usage with lung cancer.^9^ It is equally important, however, to appreciate that observational data can also produce results that are refuted when experimental trials are conducted subsequently (e.g., hormone-replacement therapy trials showing increased cardiovascular disease risk in postmenopausal women counter to previous observational data analysis^31^). Many of the most powerful observational studies capitalize on natural experiments in the data (see Step 4), and the large size of commercial app datasets often increases the chance that they include the necessary exogenous variation (e.g., weather^18^ or delays in friendship acceptance in an app^19^) to allow for natural experiments. Another goal of analyzing observational datasets is to generate hypotheses to test with rigorous experimental approaches where causal relationships can be established. For example, the observational work of Shameli et al.^20^ suggests that the gender makeup of participants in an online physical activity competition predicts how motivating the competition will be. A randomized controlled trial could subsequently test whether individuals assigned to competitions where participant gender is evenly split see greater increases in physical activity than those assigned to groups where all participants are of the same gender.

The researcher must next identify and gain access to the necessary dataset(s). Forming research-focused partnerships with industry is a valuable source of data for academic researchers. In establishing industry collaborations, we begin by talking with the potential partner to identify research goals that are of mutual interest. Another important aspect is a data sharing agreement, which spells out the rights and responsibilities of both parties. We have found that having a template for the data sharing agreement helps advance the collaboration. For example, our template includes a clause to allow for open publication of results, with attribution of the industry partner based on their preference, and we have found that companies generally accept this. Table 1 includes additional elements of our data sharing template. The partner must also ensure that the app’s or device’s terms of service permit data sharing for research, and the researcher must acquire any necessary institutional review board approvals. Ethical concerns should also be considered. For example, GPS or other location information should be obscured when possible (e.g., Strava heat maps came under fire recently for accidentally revealing the location of secret military bases abroad^32^).

**Table 1:** Elements of a data sharing agreement with an app or wearable company

Data ownership	The partner owns the dataset; the act of data sharing does not transfer that ownership to the researcher.
Scope of data use	The researcher will use the dataset only for non-commercial research and education purposes.
Data access	Access to the data within the researcher’s organization will be granted only on a need-to-have basis.
User anonymity	The researcher will not use the data to try and identify and contact the users.
Publications	The researcher has the right to publish the results with an attribution or not, as preferred by the partner, provided that the data cannot be reconstructed from the publication and that no partner’s commercial secrets are disclosed.
Licensing of results	The partner has non-exclusive, royalty-free license to any results obtained from the data

Our data sharing agreement template typically includes some or all of the following elements. You should review your data sharing agreement with the appropriate officials at your institution

**Fig. 3:**
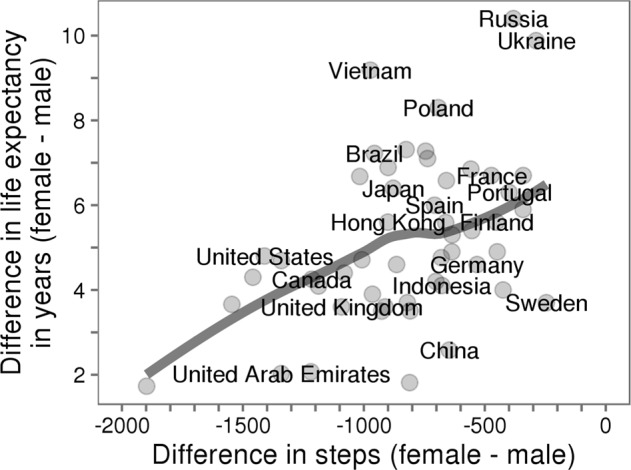
Difference in life expectancy as a function of the difference in activity volume between females and males. When the gap in steps between females and males gets smaller (i.e., less negative), females outlive males by more years (using World Bank data^65^; grey line is LOESS fit; *R*^2^ = 0.20). These results indicate that the reduced activity levels recorded by women in countries with high activity inequality may have significant implications for health. (Plot shows countries with more than 1000 subjects from the Argus dataset described in our previous work.^11^)

In many cases it is helpful to combine multiple datasets. For example, by combining data from Azumio’s activity tracking app with the World Bank’s database of life expectancy by gender in countries around the world, we were able to show that when women’s physical activity is reduced compared to men, their life expectancy is also reduced compared to men in the same country (Fig. 3). In this analysis we could not link the data individually, but in some cases this linking is possible. For example, if the location of an individual is known, you can link with weather databases to quantify how weather patterns affect an individual’s physical activity.^18^ Thus, new insights can be drawn by adding context to measurements from wearables (e.g., location information). Data collected through more traditional research studies can also complement datasets from commercial apps and wearables by helping to demonstrate that a phenomenon of interest is present across multiple populations and measurement modalities. There are a number of publicly available datasets describing physical activity, other health behaviors, and health status indicators of interest like disease status, weight, etc. We have compiled many of these.^33^

Before proceeding with your analysis, you should consider whether your research question will be answerable given the limitations of available data. In Steps 2–5, we will discuss approaches to assess and account for confounding, missing data, and selection bias, common challenges in analyzing data from wearables. But before proceeding, you should make sure your question and the challenges of the dataset do not set you up for failure. For example, if sensor measurements are known to be imperfect, trends are often easier to study and gain confidence in than absolute differences. Individuals from developing countries and individuals with low socioeconomic status are underrepresented in many datasets; thus, questions focused on these groups may thus be better addressed with a different type of study.

### Step 2: prepare the data for analysis

The next step is to prepare your data for analysis, defining and implementing strategies to handle issues such as inaccuracies in the sensor data, missing data, or outliers. The process of preparing your data typically comprises several steps.

*Characterize the accuracy and precision of sensors and determine if appropriate for your research question(s)*. You can use literature that describes sensor accuracy to determine if any wearables or apps used in the dataset have been previously validated.^10,34,35^ If existing literature is insufficient, you should conduct independent experiments to test accuracy. For example, you might need to test the app or device in a new population of interest (e.g., individuals with osteoarthritis) or for a new health behavior/activity of interest. In some cases, if sensor accuracy is low, precision may be sufficient (e.g., if trends are most important to your research question).

**Fig. 4:**
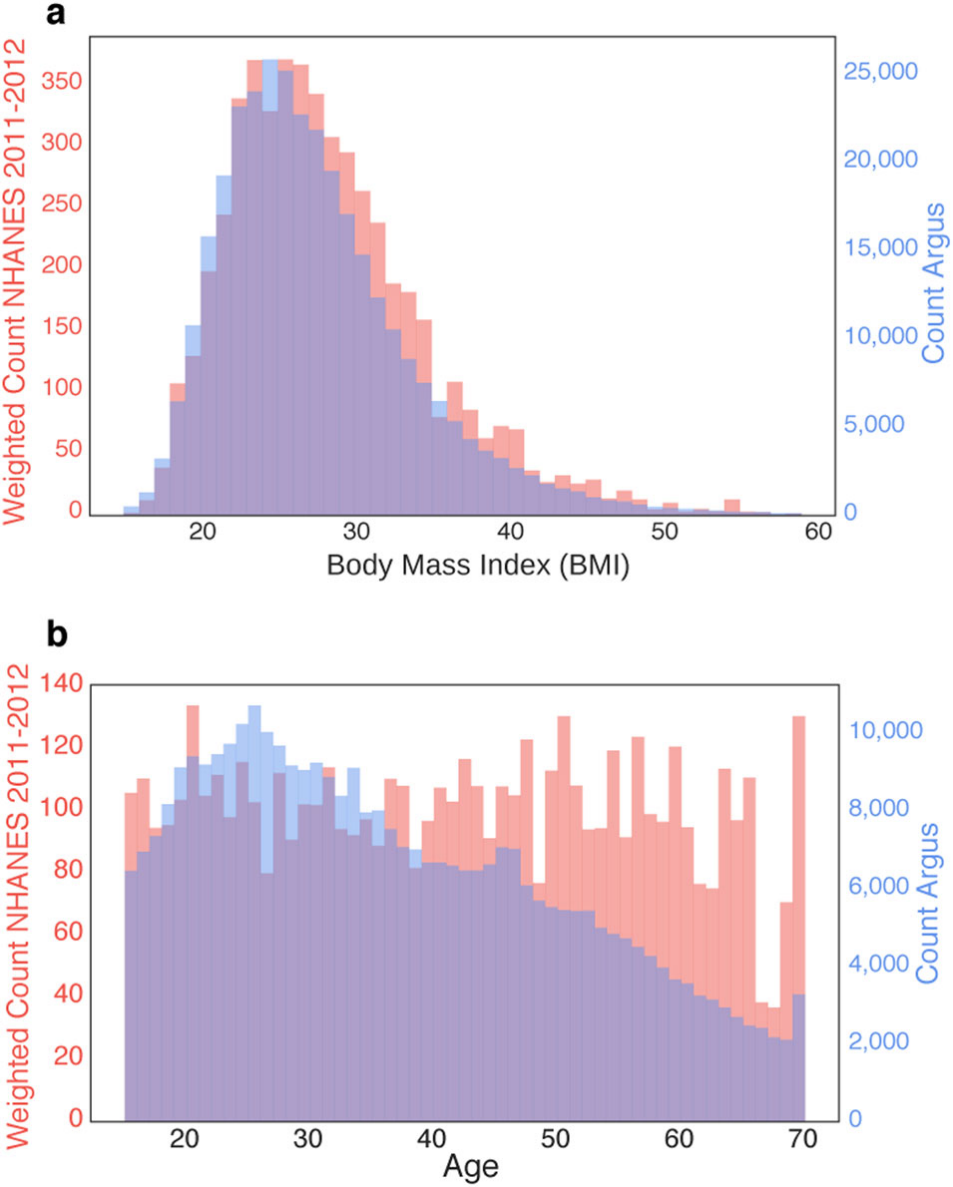
Comparison of demographics of U.S. users of a smartphone and data from traditional surveillance studies. **a** Body Mass Index (BMI) distribution of users of the Argus app (blue) vs. the U.S. population as measured in the National Health and Nutrition Examination Survey^66^ (NHANES; red). **b** Age distribution of users of the Argus app vs. NHANES. The counts for the NHANES sample are weighted according to the NHANES-provided sample weights, thus the distributions approximate the general U.S. population and the total of the weighted counts in the histogram matches the number of individuals in the 2011–2012 NHANES study year. While there are differences between the distributions, the app dataset, due to its massive size, has large coverage of users between the ages of 15 and 70 and BMIs from 20 to 40. For example, the dataset includes 32,000 individuals in the U.S. over age 60 and 113,000 individuals in the U.S. whose BMI classifies them as obese

*Define the population of interest and determine if the population is sufficiently represented*. The population you study is driven by your research question (e.g., are you focusing on elderly individuals or obese individuals?). While, in relative terms, a particular demographic subgroup may be underrepresented in a dataset, in absolute terms the actual sample size of that subgroup may be more than sufficient to answer useful questions of interest. For example, older adults may be underrepresented compared to the general population, but if the sample size is still large (Fig. 4), this does not preclude you from researching relationships in this group. If the goal is to answer a question about the general population, you may wish to resample to match the population of interest (e.g., to achieve a known gender ratio or distribution of ages). Alternate strategies to resampling are discussed in Step 5.

*Clean the data to remove outliers and erroneous values*. Begin by inspecting the data, examining the range of each value to determine a definition for outliers. This is typically done at the discretion of the researcher based on expected values (e.g., what is a reasonable maximum body weight or steps taken in a day for an adult?) or by defining a threshold based on standard deviations from the mean. Similarly, Helander et al.^16^ created thresholds for weight change between measurements to eliminate cases where multiple individuals used the same scale or the user weighed their suitcase. You should also examine the distribution of data, since peaks and discontinuities can reveal problems. For example, people might not change default values for height and weight in the app. You should also ensure that all the data are in the same units and that the dataset does not include duplicate entries (e.g., as performed in the work of Serrano et al.^24,25^).

*Characterize missing data, create thresholds for inclusion, and define approaches for handling missing data*. Data can be missing for a variety of reasons; for instance, a day could be missing because the individual forgot her smartphone at home or an hour of recording could be missing because an individual did not want to wear his activity tracker to a formal event. Users of apps can also neglect to enter demographic information of interest, such as height or weight.

As a researcher, you should define thresholds for how much health behavior data (e.g., activity or sleep) is required for a day or recording session to be included for analysis, a user to be included, and a group of interest to be included. In some cases, the thresholds can be informed by the literature (e.g., many accelerometer studies of physical activity only use subjects with at least three days of recordings based on an analysis by Tudor-Locke et al.^36^). If the literature does not provide guidance, you should choose reasonable thresholds and ensure you reproduce known trends (see Step 3) and conclusions are robust to decisions made (see Step 5). For example, in our study of activity inequality,^11^ we chose to include countries with at least 1000 users, but found that moderate increases or decreases in this threshold did not affect the main conclusions of our work.

We commonly want to examine health behavior data along with demographics or other covariates. If your dataset is sufficiently large and you can demonstrate that individuals with missing demographic and other covariate data are the “same” as individuals without missing data, you might require complete cases for analysis. One approach for demonstrating two populations are sufficiently similar in a set of relevant variables is to use standardized mean difference (SMD). The SMD is defined as the difference in the means of treated and control groups (in this case, missing and not missing data) divided by the overall standard deviation.^37^ Covariates with absolute SMD lower than 0.25 are considered balanced. Note that SMD is preferred over hypothesis tests and p-values as a measure of balance since the latter conflate changes in balance with changes in statistical power. If there are differences between individuals or days with and without missing data or the dataset is not large enough to support a complete case analysis, imputation can be used.^38,39^ Another approach is descriptive analysis of the selection effect (i.e., quantifying that subjects with data were X much younger and Y more active than subjects that were excluded for missing data).

### Step 3: verify that the datasets reproduce previously published datasets and analyses

**Fig. 5:**
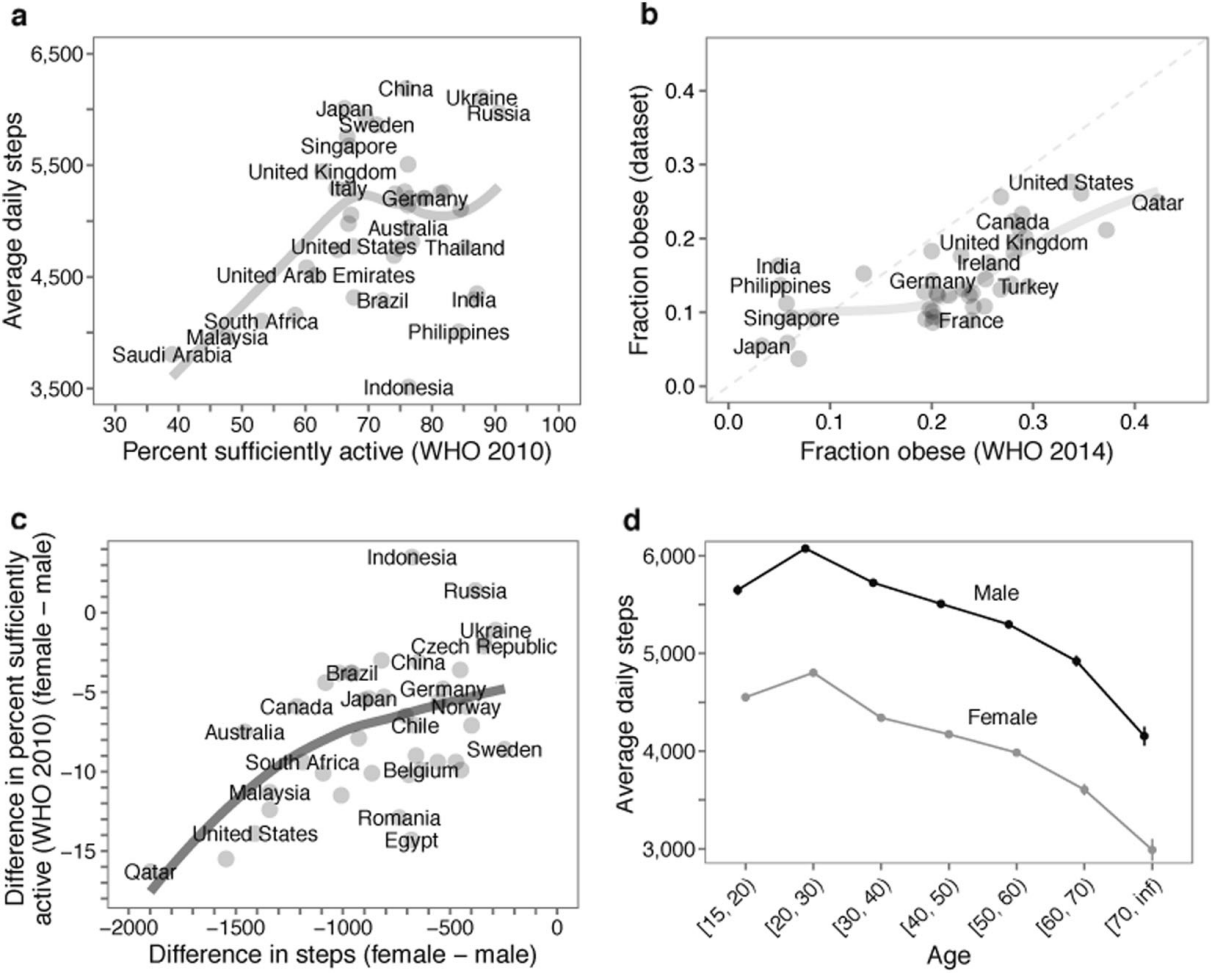
Verifying that a smartphone app dataset reproduces previously reported relationships between physical activity, geographic location, age, and gender. In our study of activity inequality,^11^ we conducted extensive analyses comparing the app dataset to previously published datasets. **a** WHO physical activity measure^67^ versus smartphone activity measure (LOESS fit). The WHO measure corresponds to the percentage of the population meeting the WHO guidelines for moderate to vigorous physical activity based on self-report. The smartphone activity measure is based on accelerometer-defined average daily steps. We found a correlation of *r* = 0.3194 between the two measures (*P* < 0.05). Note that this comparison is limited because there is no direct correspondence between the two measures—values of self-reported and accelerometer-defined activity can differ,^40^ and the WHO confidence intervals are very large for many countries. **b** WHO obesity estimates^68^ based on self-reports to survey conductors, versus obesity estimates in our dataset, based on height and weight reported to the activity-tracking app (LOESS fit). We found a significant correlation of r = 0.691 between the two estimates (*P* < 10^−6^). **c** Gender gap in activity estimated from smartphones is strongly correlated with previously reported estimates based on self-report (LOESS fit). We found that the difference in average steps per day between females and males is strongly correlated to the difference in the fraction of each gender who report being sufficiently active according to the WHO (*r* = 0.52, *P* < 10^−3^). **d** Daily step counts are shown across age for all users. Error bars correspond to bootstrapped 95% confidence intervals. Observed trends in the dataset are consistent with previous findings; that is, activity decreases with increasing BMI^69–71^ and is lower in females than in males.^70,72–74^ This figure is adapted from our previous work^11^ and reproduced with permission

Next, you must verify that your new dataset(s) are able to reproduce previous results. The aim here is to establish convergent construct validity: are you measuring what you think you’re measuring? You should review similar literature and publicly available datasets that overlap with your dataset. If the literature or analysis of gold-standard datasets point to consistent conclusions (e.g., about the relationships between gender and activity and sleep), you should determine if your dataset produces these same relationships. If this analysis identifies conflict, there may be reasonable explanations for the difference. For example, traditional surveillance studies have their own limitations, such as bias due to self report,^40^ so comparing activity levels across different age groups might show different magnitudes, but similar trends (Fig. 5). If comparison with previous results reveals differences, you may also need to reassess the parameters and thresholds determined in Step 2. You will also conduct sensitivity and robustness analysis after analyzing the data to answer your research question of interest (Step 5).

An orthogonal and equally important approach is showing discriminant construct validity, where the aim is to demonstrate that your sensor or dataset does not measure things it shouldn’t. For example, in our analysis of the dataset from Azumio, we showed that there was no correlation between the average number of daily steps within a country and the average estimated weartime.^11^ Thus our steps count was not erroneously measuring simply how much individuals were using their phones.

### Step 4: analyze the data to answer your (new) research question

The focus of this paper is analyzing data that have already been collected (i.e., observational data) and the topic of observational study design has been reviewed (e.g.,^41,42^). Thus, we will highlight areas of relevance to health app and device datasets.

**Fig. 6:**
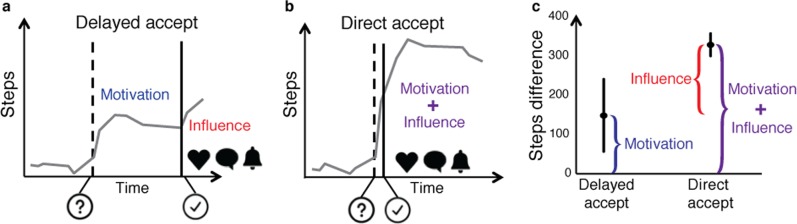
Example of a natural experiment using observational data from a smartphone app for tracking activity. The Argus smartphone app (Azumio, Inc.), includes a social network that users can opt to join. Althoff and colleagues^19^ sought to uncover if and how forming social connections affects social activity. Since users who join and are active in the social network may be more intrinsically motivated to increase their activity, they used a natural experiment to isolate the effects of social influence from other factors that could influence activity. In particular, they compared the change in activity between **a** individuals who sent out a friend request (question mark) that was immediately accepted (check mark) and **b** individuals whose friend request was not accepted for more than 7 days. Note the curves in **a** and **b** are for illustrative purposes and do not represent actual subjects. Once a friendship is accepted, the user receives notifications of their connections’ activities (e.g., going for a run), and can comment on their connections’ activity posts (denoted by the heart, text box, and notification bell in **a** and **b**). Since the two groups were similar in all aspects except whether their friend request was accepted within 7 days, the additional increase in activity of the direct acceptance group can be attributed to social influence. **c** This social influence resulted in users taking 400 more steps per day on average. Error bars indicate bootstrapped 95% confidence intervals

One common goal and challenge in observational data analysis (and all of science!) is moving beyond correlations and establishing causal relationships. In observational datasets collected from commercial apps and wearables, individuals are not assigned to different treatments (e.g., living in a city with high or low walkability) at random, as in randomized controlled trials. Instead, someone who is more motivated to be active may choose to live in a city that is more walkable. One approach to counter the confounding this can create is to identify natural experiments in the available data. In a natural experiment, the researcher finds a case or cases in the dataset where exposure to the treatment of interest is governed by circumstances that have not been manipulated by the researcher but can be argued to be random. While these natural experiments are rare and can be hard to identify, the datasets from commercial apps and wearables are large enough that these “rare” occurrences happen in sufficient numbers. For example, Althoff et al.^19^ used a delay in the acceptance of a friend request in a health app’s social network feature to separate the effects of increased intrinsic motivation to be active from the boost in activity resulting from a new friendship (Fig. 6). Similarly, Aral and Nicolaides^18^ capitalized on global weather variation and geographically distinct friendships to show that exercise is contagious and the level of contagion varies based on gender and relative levels of activity. Other natural experiments we might capitalize on are geographic relocation (e.g., between cities with higher or lower walkability), transit strikes, or sudden changes in air quality. You must argue and present as much evidence as possible that your natural experiment approximates random assignment. For example, in the study by Althoff et al.^19^ they showed that 22 variables (e.g., age, BMI, and previous activity levels) were balanced (SMD < 0.25) between the groups with and without a delayed friend acceptance.

**Fig. 7:**
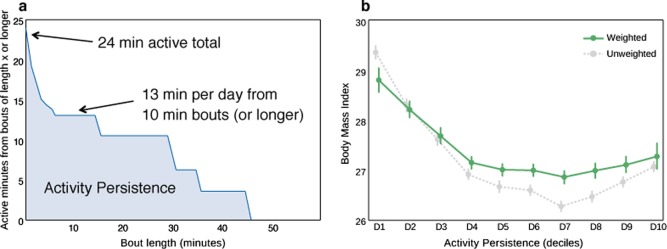
Example of propensity scoring to isolate the effects of a treatment that comes in different doses (physical activity) from other confounding factors. The Argus smartphone app (Azumio, Inc.) collects minute by minute step counts. **a** For each user, we can construct a plot of activity bout length (X) vs. the average number of minutes per day spent in activity of at least X minutes. We call the area under this curve an individual’s activity persistence. In the figure we include users with at least 10 days of step tracking data. **b** We next want to understand how activity persistence influences quantities like BMI. Since individuals with higher or lower activity persistence may be different in other ways that influence BMI (such as age and gender), we used inverse probability of treatment weighting (IPTW) to isolate the effects of activity. The grey curve shows the BMI of individuals in each decile of activity persistence (where higher deciles indicate more bouts of longer duration), without any weighting. The green curve shows the relationship after we have used IPTW to minimize the influence of other factors like age and gender on the estimated BMI for each decile of activity persistence. Error bars correspond to bootstrapped 95% confidence intervals

Another approach to address the fact that exposure to different “treatments” (e.g., levels of physical activity) is not assigned at random in observational datasets is to build and apply a model that estimates the propensity of an individual to receive treatment based on measured covariates. These propensity scores can be used for matching, stratification, weighting, or covariate adjustment.^43^ Propensity scoring is well-established in the case of binary treatments (e.g., they match individuals in the “treatment” population to comparable individuals in the control population) and several papers review the topic.^42–44^ There is evidence that a weighting approach reduces bias when estimating treatment effects; however, along with covariate adjustment, weighting may be more sensitive to whether the propensity model is correctly specified.^43^ In all cases, it is vital to assess whether the propensity model adequately achieves balance (e.g., using SMD) between covariates for the treated and untreated conditions.^43^ We have also explored approaches for propensity weighting in the case where the treatment of interest comes in varying doses. In particular, we were interested in using data for the Argus smartphone app to understand how varying activity levels were related to BMI and other health indicators. Since app users who take more and less steps are generally different in other ways that may also affect our outcome variables of interest, we extended inverse probability of treatment weighting to estimate a dose-response relationship while controlling for these confounding factors (Fig. 7). With this analysis we found that longer bouts of activity are associated with lower BMI and that even relatively short bouts—of 5 or 10 minutes—are associated with better health measures.

There are several other approaches for moving toward causality—some are relatively new and others have a long history of use in econometrics, but have not yet been widely applied to large-scale commercial health behavior datasets. Instrumental variables (which were first explored in the 1920s) are a method for analyzing natural experiments, where researchers capitalize on a covariate that affects the outcome variable of interest only by its influence on the explanatory variable of interest.^45^ For example, state cigarette taxes have been used as an instrument to relate maternal smoking to birth weight.^46^ In the case of health behavior datasets from commercial apps and wearables, we might use distance from a newly built public park or transit stop as an instrumental variable to study how environment affects activity. Another classic technique from econometrics is regression discontinuity.^47^ In this design, the investigator capitalizes on treatments prescribed above and below a threshold (e.g., a scholarship awarded to individuals with an SAT score of above 1500) to argue that differences (e.g., in college graduation rates) between individuals just above and just below the threshold can be primarily attributed to the treatment. In the case of commercial apps, this approach might be used to gauge whether a threshold-triggered notification or message (e.g., to stand-up if sedentary time has lasted for 60 min) in an app changed an individual’s behavior. As in the case of natural experiments, the investigator must argue and present quantitative evidence where possible to show that the instrumental variable or regression discontinuity is reasonable. Doubly robust estimators are a relatively new approach to extract causal evidence from observational datasets. A regression model is one widely used approach to try to handle confounding in observational datasets, but it must be correctly specified to estimate a treatment effect. The propensity score models discussed in the previous paragraph must also be correctly specified. Doubly robust estimators^48^ estimate the treatment effect while only requiring one of these models be correctly specified.

Another challenge with wearable datasets is the massive scale of the data; datasets can have millions or even billions of samples. Such datasets might be too large to be easily analyzed with standard desktop or laptop computers. Often, only a subset of input fields are relevant for the study. In those cases, the data can be preprocessed with only the relevant fields being extracted, which can yield a significantly smaller dataset. If the resulting dataset is still too large for a personal computer, the next option is a large memory compute server. These computers offer similar computing environments and analysis tools (e.g., Python or R) as personal computers, and they can provide orders of magnitude more main memory than personal computers. While an in-house large memory compute server might be prohibitively expensive, all major cloud providers offer on-demand machines with several terabytes (TBs) of main memory, which is sufficient for all but the largest datasets. If the dataset size exceeds even the capabilities of the large memory servers, then a distributed approach is needed, where multiple machines work together on a single task. Distributed approaches can be scaled up by adding more machines to accommodate the size of the dataset being analyzed. These approaches require modifications to the analysis scripts and programs to take advantage of the distributed capabilities. Common environments for distributed analysis are Hadoop^49,50^ and Spark,^51,52^ available as open source or commercial offerings and supported by major cloud providers.

### Step 5: check robustness of conclusions

You must now determine whether your conclusions are robust. The goal in this step is to try to prove yourself wrong. You should identify all the potential limitations and shortcomings of your dataset and approach and test whether your conclusions are still valid. The essential tests should establish internal validity (i.e., are your research design and methods sound?) and external validity (i.e., can your findings be generalized to other people and/or situations?). To establish internal validity, several questions must be addressed.

*Have you accounted for selection bias and confounding?* If your analysis aimed to establish a causal relationship, your study design (Step 4) should account for potential confounders, and any potential unmeasured confounders should be acknowledged. If your analysis involved mining the data for new clusters and correlations, it is vital to identify potential confounders and determine how they may have influenced the results. For example, say you find clusters of less engaged and highly engaged app users and the highly engaged users show greater weight loss. If the highly engaged users tend to be younger, without further analysis you cannot infer that engagement with the app leads to weight loss.

*Have you accounted for any multiple hypothesis testing?* Testing multiple hypotheses increases the risk of Type I (false positive) errors and you must use an appropriate technique to account for this. A simple approach is the Bonferroni correction, which adjusts the study-wide significance level based on the number of hypotheses tested. Benjamini and Hochberg^53^ also provide a method for selecting a subset of rejected null hypotheses that achieves a desired false discovery rate (where the false discovery rate is the ratio of false positives to all positives). The second approach has greater power, but may increase the rate of false positives.^54^

*Are any distributional or parametric assumptions valid?* Parametric statistical tests and many statistical models rely on distributional or other assumptions. In some cases, these assumptions are justified. In the app data we have analyzed, we have found, for example, that steps per day tends to be similar to a normal distribution (with some truncation since steps per day can’t be negative) and the number of connections in a social network to follow a power law distribution. If assumed distributions do not hold in your data, you should choose non-parametric tests or be able to argue why your choice of test or model is still reasonable (e.g., drawing on previous literature that shows non-normality is acceptable if sample sizes are large).

*Are your findings robust to analysis choices you made in Steps 2 and 5?* In Step 2, you chose an approach to handle missing data and outliers. You should verify that your conclusions are similar if you use another valid approach. For example, you could compare analyses using complete case analysis to an imputation approach. You should also examine how removing and including outliers and varying thresholds for outliers affect your results.

If conclusions are sensitive to an analysis choice or limitation of your dataset, you may need to revisit your research question because the limitations of the dataset may prevent answering it. If you test new hypotheses, as pointed out above, you must take precautions against false discovery and p-hacking.^55^ Another option is to find or collect additional data (e.g., you could collect a small, targeted set of data prospectively).

Alternately, if the sensitivity is minor or explainable, you can report the sensitivity as a limitation when publishing results. Many scientists are skeptical about using commercial app datasets and this skepticism is merited. However, if sensitivities and limitations have been thoroughly examined and documented, the inherent “messiness” of such datasets should not preclude their publication. Traditional surveillance and experimental studies of health behaviors have limitations (e.g., small sample sizes and bias due to self-report), and consumer wearable and app data can complement traditional approaches to move the field forward.

**Fig. 8:**
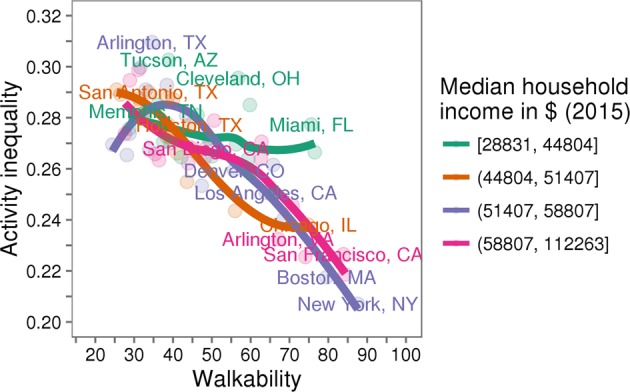
Relationship between walkability and activity inequality holds within cities in the USA of similar income. We found that walkability was associated with lower levels of activity inequality. To help account for potential confounding due to socioeconomic factors, we grouped the 69 cities in our analysis into quartiles based on median household income (data from the 2015 American Community Survey^75^). We found that walkable environments were associated with lower levels of activity inequality for all four groups (LOESS fit). The effect appears to be attenuated for cities in the lowest median household income quartile. These results suggest that our main result—activity inequality predicts obesity and is mediated by factors of the physical environment—is independent of potential socioeconomic bias in our sample. This figure is adapted from our previous work^11^ and reproduced with permission

To establish external validity, you must identify the populations and situations to which your findings apply. Given the nature of the data from consumer wearables and apps, missing data is common, and since the population is typically a convenience sample, it may not be a match to the population of interest. For example, one challenge of analyzing data from apps and wearables is that the population of users is likely skewed towards individuals of higher socioeconomic status than the general population. In our study of activity inequality, we found that walkability was a key predictor of low activity inequality. We had no way to measure the socioeconomic status of users; however, we were able to show that high city walkability was associated with low activity inequality across U.S. cities with a range of median income levels (Fig. 8).

Another useful technique for assessing the robustness and usefulness of your results is to compare any models you have built to a simple or null model. This comparison will help you assess whether model predictive power is meaningful beyond simpler explanations that are encoded in a null model. For example, in the case of network analysis, the null model is a graph that is random, but maintains the structural features of your network. In a social network, where edges represent friendships, the edges can be rewired randomly while preserving the degree of each node and thus the number of friends of each person.^56^ Comparing your model to this null model will help you to assess whether model predictive power is meaningful or just a property of any similar random network.

Examining *p*-values is a common approach in biomedical research, but in very large datasets many relationships are statistically significant. Even a relationship with a small effect size can have very low *p*-values in large samples. Thus, we find that “practical significance” often matters more. Even if a relationship is statistically significant, is the effect size still large and meaningful? How much variance in the data is explained by a particular relationship? These quantities should be reported in addition to, or even instead of, p-values. In the case where you are building a predictive model, cross-validation or other approaches to testing the model on a reserved test or hold-out dataset (not used to train the model) should show, for example, sensitivity and specificity values that indicate the model will be useful in practice. Testing a model on an entirely different dataset is rarely done but is valuable for evaluating a model’s generalizability.

### Step 6: share results

In the final step, you should document the dataset, methods, and results for publication, with the goal of adding to our knowledge about health behaviors and inspiring new research. You should be as transparent as possible, documenting limitations of your analysis and describing all the analyses in Steps 2–5 to draw your conclusions and establish their validity. In conjunction with publication, we encourage researchers to share their models, software, datasets, and other digital resources wherever possible. You must consider what data is ethical to share publicly while still protecting the identity of users; in some cases, sharing aggregated data is safest. Taking the extra time to fully document and share your approach, results, and code allows others to reproduce and extend your results, completing the circle of Fig. 2 and increasing the impact of your research.

## Future research: challenges and opportunities

Access to data remains a significant barrier to research. Vast amounts of data have been and are being collected by smartphones and wearables, but they are largely siloed at individual companies. A few strategies might unlock more of these data for analysis by researchers to uncover new ways to monitor and improve health. We encourage users to release anonymized data to advance research and, at minimum, there should be better means for users to download their own data and donate it for research, if they wish to do so. Going a step further, researchers can also leverage the growing movement toward citizen science, where researchers engage individuals in the process of collecting and analyzing data and then employing the results to effect changes in their community.^57^ For example, Rodriguez and colleagues^58^ recently showed that using a citizen science mobile app called the Discovery Tool (which allows participants to take geo-coded photos and describe barriers to and enablers of healthy living in their local environments) increased engagement in a safe routes to school program along with rates of walking and biking to school. To protect user privacy, we need better standards and algorithms for anonymizing activity data. Government, health care, academic, and industry partnerships and consortiums around app and wearable data sharing and analysis are also needed.

The research community needs more gold-standard population level data against which to compare. Initiatives like the UK Biobank^59^ have conducted large cohort studies that provide a valuable resource, including wearable data, along with medical records, imaging, genome, and other measures. Another area of interest is identifying the relationships between activity, sedentary time, and sleep over the full 24-hour day. Data from commercial apps and wearables could provide insights into these relationships, but better algorithms and sensors are needed to accurately differentiate between these activities. Additionally, the research community needs transparency. Understanding how algorithms are computing quantities of interest—like steps or activity counts—is vital for researchers to understand sensitivities and limitations of their datasets. We encourage companies to share the details of their algorithms with researchers when possible.

To both increase trust and continue to advance our knowledge, we also need new methods for working with large-scale observational datasets. For example, natural experiments, instrumental variables, and regression discontinuities are powerful approaches for helping establish causality, but their discovery largely relies on the ingenuity of the research team. Approaches to automatically identify these natural experiments^60^ could help us discover more insights about the many factors drive physical activity and health. We also need better tools to analyze and communicate uncertainty. For example, automated tools to screen for potential confounding would be highly valuable. And in most datasets, there are many, or even infinite, choices for thresholds, tuning parameters, combinations of features, removing outliers, etc. These choices collectively span a “multiverse” of analyses^61^ and reflect “researcher degrees of freedom”.^62^ We need better tools to quantify, communicate, and visualize this uncertainty.

We hope there will continue to be new collaborations between biomedical researchers and data scientists, where cross-disciplinary expertise is needed to tackle some key challenges. For example, “just-in-time” interventions, developed with insights from health behavior change and data science experts, could optimize prompts and nudges for health behavior change at the individual, group, and population levels. Collaboratively tackling these and other challenges will help commercial apps and devices have a sustained, positive impact on public health.

## References

[1] Fox, S. Duggan, M. *Tracking for health*. Pew Research Internet Project (2013). http://www.pewinternet.org/2013/01/28/tracking-for-health.

[2] Digital Health Market by Growth Prospects, Trends, Share, Growth, Forecast by 2017−2025. https://www.transparencymarketresearch.com/digital-health-market.html. Accessed 31 Oct 2018.

[3] World Health Organization. *Global recommendations on physical activity for health*. (WHO, 2010). 26180873

[4] Biswas A (2015). Sedentary time and its association with risk for disease incidence, mortality, and hospitalization in adults: a systematic review and meta-analysis. Ann. Intern. Med..

[5] St-Onge M-P (2016). Sleep duration and quality: impact on lifestyle behaviors and cardiometabolic health: a scientific statement from the american heart association. Circulation.

[6] United Nations Secretary General. *Prevention and control of non-communicable diseases*. (United Nations, 2011).

[7] Cadmus-Bertram LA, Marcus BH, Patterson RE, Parker BA, Morey BL (2015). Randomized trial of a Fitbit-based physical activity intervention for women. Am. J. Prev. Med..

[8] Wharton CM, Johnston CS, Cunningham BK, Sterner D (2014). Dietary self-monitoring, but not dietary quality, improves with use of smartphone app technology in an 8-week weight loss trial. J. Nutr. Educ. Behav..

[9] Bunn JA, Navalta JW, Fountaine CJ, Reece JD (2018). Current state of commercial wearable technology in physical activity monitoring 2015–2017. Int. J. Exerc. Sci..

[10] Evenson KR, Goto MM, Furberg RD (2015). Systematic review of the validity and reliability of consumer-wearable activity trackers. Int. J. Behav. Nutr. Phys. Act..

[11] Althoff T (2017). Large-scale physical activity data reveal worldwide activity inequality. Nature.

[12] Atkinson AB (1970). On the measurement of inequality. J. Econ. Theory.

[13] Walch OJ, Cochran A, Forger DB (2016). A global quantification of “normal” sleep schedules using smartphone data. Sci. Adv..

[14] Althoff, T., Horvitz, E., White, R. W. & Zeitzer, J. Harnessing the Web for Population-Scale Physiological Sensing: A Case Study of Sleep and Performance. In *Proc. of the 26th International Conference on World Wide Web*, 113–122 (International World Wide Web Conferences Steering Committee, Perth, Australia, 2017).

[15] Kim K-I (2018). Real world home blood pressure variability in over 56,000 individuals with nearly 17 million measurements. Am. J. Hypertens..

[16] Helander EE, Wansink B, Chieh A (2016). Weight gain over the holidays in three countries. N. Engl. J. Med..

[17] Howell, P. D. et al. Analyzing Taste Preferences From Crowdsourced Food Entries. In *Proc. of the 6th International Conference on Digital Health*, 131–140 (ACM, Montreal, Quebec, Canada, 2016).

[18] Aral S, Nicolaides C (2017). Exercise contagion in a global social network. Nat. Commun..

[19] Althoff, T., Jindal, P. & Leskovec, J. Online actions with offline impact: how online social networks influence online and offline user behavior. In *Proc. Tenth ACM International Conference on Web Search and Data Mining*, 537–546 (ACM, Cambridge, United Kingdom, 2017). 10.1145/3018661.3018672 PMC5361221 28345078

[20] Shameli, A., Althoff, T., Saberi, A. & Leskovec, J. How Gamification Affects Physical Activity: Large-scale Analysis of Walking Challenges in a Mobile Application. In *Proc. of the 26th International Conference on World Wide Web*, 455–463 (International World Wide Web Conferences Steering Committee, Perth, Australia, 2017). 10.1145/3041021.3054172 PMC5627651 28990011

[21] Wang, Z., Derr, T., Yin, D. & Tang, J. Understanding and Predicting Weight Loss with Mobile Social Networking Data. In *Proc. of the 2017 ACM on Conference on Information and Knowledge Management*, 1269–1278 (ACM, Singapore, 2017).

[22] Althoff T, White RW, Horvitz E (2016). Influence of Pokémon Go on physical activity: study and implications. J. Med. Internet Res..

[23] Kurashima, T., Althoff, T. & Leskovec, J. Modeling Interdependent and periodic real-world action sequences. In *Proc. 2018 World Wide Web Conference*, 803–812 (International World Wide Web Conferences Steering Committee, Lyon, France, 2018). 10.1145/3178876.3186161 PMC5959287 29780977

[24] Serrano KJ, Yu M, Coa KI, Collins LM, Atienza AA (2016). Mining health app data to find more and less successful weight loss subgroups. J. Med. Internet Res..

[25] Serrano KJ, Coa KI, Yu M, Wolff-Hughes DL, Atienza AA (2017). Characterizing user engagement with health app data: a data mining approach. Transl. Behav. Med..

[26] McConnell MV (2017). Feasibility of obtaining measures of lifestyle from a smartphone app: The MyHeart Counts Cardiovascular Health Study. JAMA Cardiol..

[27] Lin, Z., Althoff, T. & Leskovec, J. I’ll Be Back: On the Multiple Lives of Users of a Mobile Activity Tracking Application. In *Proc. 2018 World Wide Web Conference*, 1501–1511 (International World Wide Web Conferences Steering Committee, Lyon, France, 2018). 10.1145/3178876.3186062 PMC5959281 29780978

[28] Park, K., Weber, I., Cha, M. & Lee, C. Persistent Sharing of Fitness App Status on Twitter. In *Proc. 19th ACM Conference on Computer-Supported Cooperative Work & Social Computing*, 184–194 (ACM, San Francisco, California, USA, 2016).

[29] Sperrin M (2016). Who self-weighs and what do they gain from it? A retrospective comparison between smart scale users and the general population in England. J. Med. Internet Res..

[30] Nelson MB, Kaminsky LA, Dickin DC, Montoye AHK (2016). Validity of consumer-based physical activity monitors for specific activity types. Med. Sci. Sports Exerc..

[31] Rossouw JE (2002). Risks and benefits of estrogen plus progestin in healthy postmenopausal women: principal results from the women’s health initiative randomized controlled trial. JAMA.

[32] Kwai, I. What he did on his summer break: exposed a global security flaw. *The New York Times* (2018). https://www.nytimes.com/2018/01/30/world/australia/strava-heat-map-student.html.

[33] Mobilize Center. *Data Sources*, http://mobilize.stanford.edu/data-sources/. (Accessed 31 Oct 2018).

[34] Case MA, Burwick HA, Volpp KG, Patel MS (2015). Accuracy of smartphone applications and wearable devices for tracking physical activity data. JAMA.

[35] Shcherbina A (2017). Accuracy in wrist-worn, sensor-based measurements of heart rate and energy expenditure in a diverse cohort. J. Pers. Med..

[36] Tudor-Locke C (2005). How many days of pedometer monitoring predict weekly physical activity in adults?. Prev. Med..

[37] Stuart EA (2010). Matching methods for causal inference: a review and a look forward. Stat. Sci..

[38] Rubin, D. B. *Multiple Imputation for Nonresponse in Surveys* (Wiley, New York, 1987).

[39] Sterne JAC (2009). Multiple imputation for missing data in epidemiological and clinical research: potential and pitfalls. BMJ.

[40] Prince SA (2008). A comparison of direct versus self-report measures for assessing physical activity in adults: a systematic review. Int. J. Behav. Nutr. Phys. Act..

[41] Rosenbaum, P. R. *Observational Studies*. (Springer, New York, 2002).

[42] Rosenbaum PR, Rubin DB (1983). The central role of the propensity score in observational studies for causal effects. Biometrika.

[43] Austin PC (2011). An Introduction to propensity score methods for reducing the effects of confounding in observational studies. Multivar. Behav. Res..

[44] Austin PC, Stuart EA (2015). Moving towards best practice when using inverse probability of treatment weighting (IPTW) using the propensity score to estimate causal treatment effects in observational studies. Stat. Med..

[45] Angrist JD, Krueger AB (2001). Instrumental variables and the search for identification: from supply and demand to natural experiments. J. Econ. Perspect..

[46] Permutt T, Hebel JR (1989). Simultaneous-equation estimation in a clinical trial of the effect of smoking on birth weight. Biometrics.

[47] Imbens GW, Lemieux T (2008). Regression discontinuity designs: a guide to practice. J. Econom..

[48] Bang H, Robins JM (2005). Doubly robust estimation in missing data and causal inference models. Biometrics.

[49] Apache Hadoop. *Welcome to Apache Hadoop*, https://hadoop.apache.org/. (2016).

[50] Shvachko, K., Kuang, H., Radia, S. & Chansler, R. The Hadoop Distributed File System. In *IEEE 26th Symposium on Mass Storage Systems and Technologies*, 1–10 (IEEE, Incline Village, Nevada, USA, 2010).

[51] *Apache Spark™ - Unified Analytics Engine for Big Data*, https://spark.apache.org/. (Accessed 22 Oct 2018).

[52] Zaharia M (2016). Apache spark: a unified engine for big data processing. Commun. ACM.

[53] Benjamini Y, Hochberg Y (1995). Controlling the false discovery rate: a practical and powerful approach to multiple testing. J. R. Stat. Soc. Ser. B Stat. Methodol..

[54] Shaffer JP (1995). Multiple hypothesis testing. Annu. Rev. Psychol..

[55] Head ML, Holman L, Lanfear R, Kahn AT, Jennions MD (2015). The extent and consequences of p-hacking in science. PLoS Biol..

[56] Newman MEJ, Girvan M (2004). Finding and evaluating community structure in networks. Phys. Rev. E Stat. Nonlin. Soft Matter Phys..

[57] King AC, Winter SJ, Chrisinger BW, Hua J, Banchoff AW (2018). Maximizing the promise of citizen science to advance health and prevent disease. Prev. Med..

[58] Rodriguez NM (2019). Enhancing safe routes to school programs through community-engaged citizen science: two pilot investigations in lower density areas of Santa Clara County, California, USA. BMC Public Health.

[59] Sudlow C (2015). UK biobank: an open access resource for identifying the causes of a wide range of complex diseases of middle and old age. PLoS Med..

[60] Sharma A, Hofman JM, Watts DJ (2018). Split-door criterion: Identification of causal effects through auxiliary outcomes. Ann. Appl. Stat..

[61] Steegen S, Tuerlinckx F, Gelman A, Vanpaemel W (2016). Increasing transparency through a multiverse analysis. Perspect. Psychol. Sci..

[62] Simmons JP, Nelson LD, Simonsohn U (2011). False-positive psychology: undisclosed flexibility in data collection and analysis allows presenting anything as significant. Psychol. Sci..

[63] Choros Laboratory. *ScapeToad—cartogram software*, https://scapetoad.choros.ch. (Accessed 10 May 2017).

[64] Sandvik, B. *Thematic Mapping API World Borders Dataset*, http://thematicmapping.org/downloads/world_borders.php. (Accessed 10 May 2017).

[65] World Bank. *Life expectancy at birth, male/female (years)*. http://data.worldbank.org/indicator/SP.DYN.LE00.MA.IN and http://data.worldbank.org/indicator/SP.DYN.LE00.FE.IN. (Accessed 10 May 2017).

[66] Centers for Disease Control and Prevention (CDC). National Center for Health Statistics (NCHS). *National Health and Nutrition Examination Survey Data*, https://wwwn.cdc.gov/nchs/nhanes/continuousnhanes/default.aspx?BeginYear=2011.(2011–2012).

[67] World Health Organization. *Prevalence of Insufficient Physical Activity among Adults: Data by Country*, http://apps.who.int/gho/data/node.main.A893?lang=en. (Accessed 19 May 2016).

[68] World Health Organization. *Obesity (Body Mass Index≥30) (Age-Standardized Estimate): Estimates by Country*., http://apps.who.int/gho/data/node.main.A900A?lang=en. (Accessed 19 May 2016).

[69] Bassett DR, Wyatt HR, Thompson H, Peters JC, Hill JO (2010). Pedometer-measured physical activity and health behaviors in U.S. adults. Med. Sci. Sports Exerc..

[70] Bauman AE (2012). Correlates of physical activity: why are some people physically active and others not?. Lancet.

[71] Van Dyck D (2015). International study of objectively measured physical activity and sedentary time with body mass index and obesity: IPEN adult study. Int. J. Obes..

[72] Hallal PC (2012). Global physical activity levels: surveillance progress, pitfalls, and prospects. Lancet.

[73] Troiano RP (2008). Physical activity in the United States measured by accelerometer. Med. Sci. Sports Exerc..

[74] Tudor-Locke C, Johnson WD, Katzmarzyk PT (2009). Accelerometer-determined steps per day in US adults. Med. Sci. Sports Exerc..

[75] United States Census Bureau. *American Community Survey*, http://www.census.gov/programs-surveys/acs/. (Accessed 5 Oct 2016).

